# A Potential Radiomics–Clinical Model for Predicting Failure of Lymph Node Control after Definite Radiotherapy in Locally Advanced Head and Neck Cancer

**DOI:** 10.3390/medicina60010092

**Published:** 2024-01-03

**Authors:** Seunghak Lee, Sunmin Park, Chai Hong Rim, Young Hen Lee, Soon Young Kwon, Kyung Ho Oh, Won Sup Yoon

**Affiliations:** 1 Core Research and Development Center, Korea University Ansan Hospital, Ansan 15355, Republic of Korea; victor87822@gmail.com; 2 Department of Radiation Oncology, College of Medicine, Korea University Ansan Hospital, 123 Jeokgeum-ro, Danwon-gu, Ansan 15355, Republic of Korea; sunmini815@gmail.com (S.P.); crusion3@naver.com (C.H.R.); 3 Department of Radiology, Korea University Ansan Hospital, Ansan 15355, Republic of Korea; younghen@korea.ac.kr; 4 Department of Otolaryngology, Korea University Ansan Hospital, Ansan 15355, Republic of Korea; entkwon@korea.ac.kr (S.Y.K.); ohkyungho@korea.ac.kr (K.H.O.)

**Keywords:** radiomics, radiotherapy, head and neck cancer, lymph node

## Abstract

*Background and Objectives*: To optimally predict lymph node (LN) failure after definite radiotherapy (RT) in head and neck cancer (HNC) with LN metastases, this study examined radiomics models extracted from CT images of different periods during RT. *Materials and Methods*: This study retrospectively collected radiologic and clinical information from patients undergoing definite RT over 60 Gy for HNC with LN metastases from January 2010 to August 2021. The same largest LNs in each patient from the initial simulation CT (CTpre) and the following simulation CT (CTmid) at approximately 40 Gy were indicated as regions of interest. LN failure was defined as residual or recurrent LN within 3 years after the end of RT. After the radiomics features were extracted, the radiomics alone model and the radiomics plus clinical parameters model from the set of CTpre and CTmid were compared. The LASSO method was applied to select features associated with LN failure. *Results*: Among 66 patients, 17 LN failures were observed. In the radiomics alone model, CTpre and CTmid had similar mean accuracies (0.681 and 0.697, respectively) and mean areas under the curve (AUC) (0.521 and 0.568, respectively). Radiomics features of spherical disproportion, size zone variance, and log minimum 2 were selected for CTpre plus clinical parameters. Volume, energy, homogeneity, and log minimum 1 were selected for CTmid plus clinical parameters. Clinical parameters including smoking, T-stage, ECE, and regression rate of LN were important for both CTpre and CTmid. In the radiomics plus clinical parameters models, the mean accuracy and mean AUC of CTmid (0.790 and 0.662, respectively) were more improved than those of CTpre (0.731 and 0.582, respectively). *Conclusions*: Both models using CTpre and CTmid were improved by adding clinical parameters. The radiomics model using CTmid plus clinical parameters was the best in predicting LN failure in our preliminary analyses.

## 1. Introduction

Definite radiotherapy (RT) is widely used in advanced head and neck cancer (HNC) with regional lymph node (LN) metastases. Although the prognosis differs depending on the details of disease progression, loco-regional recurrence generally occurs in 10–20% for better sub-sites such as the nasopharynx or human papillomavirus (HPV)-positive oropharynx [1,2] and in up to 30–40% for worse sub-sites such as the HPV-negative oropharynx, oral cavity, larynx, and hypopharynx [3,4]. Loco-regional recurrence is quite an important issue in HNC as it not only affects survival but also affects quality of life. It ultimately requires salvage treatment.

To increase loco-regional control, an advanced RT technique has been developed. The simultaneous integrated boost (SIB) technique can add radiation doses to risky areas such as gross tumors and hypoxic regions [5,6]. Various combinations with chemo-regimen and schedule have been studied [7]. As a part of the treatment to control HNC by preserving function, induction chemotherapy is of great interest [8]. However, whether the response could be assessed in advance remains unclear. If the response prediction in the middle of definite RT is judged to be radio-resistant, early surgical resection could be considered before fibrosis occurs due to full-dose radiation. Alternatively, clinical trials on additional chemotherapy for high-risk groups with incomplete response after definite RT could also be attempted. As a result, it would be possible to perform a multidisciplinary approach in a more comprehensive way.

Radiomics is a research method that can extract various features from images using detailed image analysis, convert phenotypes into numerical values, and predict certain results. A study including advanced HNC has shown that overall survival, progression-free survival, and local control can be well predicted with radiomics features extracted from computer tomography (CT) [9]. Another study has shown that radiomics features are as good as clinical factors for predicting disease-free survival and successfully dividing patients into low- and high-risk groups [10]. Although these previous studies have shown the potential of radiomics as an imaging biomarker, both studies were conducted based on work-ups performed before definite therapy. RT could cause various tumor microenvironment (TME) changes in response to radiation [11]. However, studies focusing on TME changes during definite RT are limited. We hypothesize that intrinsic resistance could be predicted more accurately than previous methods if TME changes during RT could be reflected in radiomics.

Thus, this study evaluated the applicability of an LN failure model with radiomics extracted from different periods of initial CT images and other CT images in the middle of RT in advanced HNC with LN metastases. The improvement in each model after adding clinical parameters was then examined. An optimal model of LN failure was then suggested.

## 2. Materials and Methods

### 2.1. Patient Information

This study was conducted on patients who underwent definite RT for HNC with LN metastases from January 2010 to August 2021. The inclusion criteria were as follows: (1) HNC was initially confirmed with a pathologic evaluation; (2) LN metastases were confirmed with structural or functional images with a diameter of the short axis > 7 mm; (3) fractionated conventional RT or concurrent chemoradiotherapy (CRT) was planned; (4) an Eastern Cooperative Oncology Group performance score of 0 or 1; and (5) image sets of simulation CT were acquired before and during RT. The exclusion criteria were as follows: (1) an LN was excised before RT or infiltrated to the skin; (2) HNC originated from the skin, paranasal sinus, salivary gland, or unknown primary site; (3) the second simulation CT was delayed over one week compared with the planned schedule; (4) RT was incompletely finished with a dose less than 60 Gy; and (5) patients who had arbitrarily follow-up loss within 2 years. This study was approved by the Institutional Review Board (K-2021AS0138). Written informed consent was waived due to the retrospective nature of this study.

### 2.2. CT Imaging and Radiotherapy

**Figure 1:**
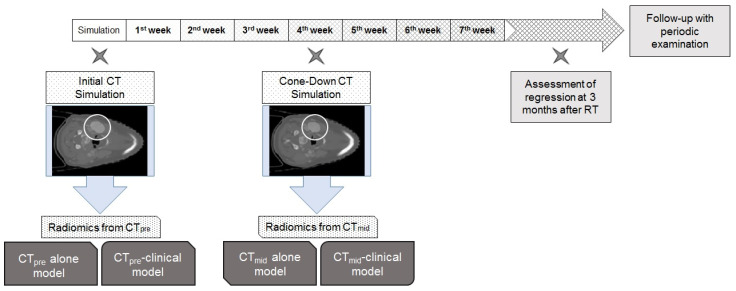
Diagram of the sequence of this study. Initial simulation CT (CTpre) and cone-down simulation CT were acquired prior to RT and in the middle of RT at 4 weeks, respectively. Lymph node within the white circle indicated the region of interest in this study.

Our institutional principle of CT simulation of HNC had generally been unchanged in the study period. After laying the patients down with a suitable headrest and fixing them with aquaplast to cover from head to shoulder, the patients’ images were acquired using a Big Bore CT simulation (Philips Medical System, Amsterdam, The Netherlands). The thickness of the CT image was 3 mm for 3D conformal RT (3DCRT) and 2 mm for intensity-modulated RT (IMRT). Iodine contrast (70 mL) for CT was injected at 1 mL/s. Images were taken 70 s after injection. After the initial plan for 3DCRT, cone-down was performed at about 40 Gy and 60 Gy excluding low- and intermediate-risk areas, respectively. High-risk areas including at least a margin of 3 mm from the gross primary tumor and LN were irradiated at up to 70 Gy. For IMRT, the same SIB technique of 2.2 Gy and 2 Gy per fraction, cone-down was performed at about 44 Gy. Low-, intermediate-, and high-risk areas were irradiated with 40 Gy, 64 Gy, and 70.4 Gy, respectively. We collected a pretreatment set of CT images (CTpre) and a mid-treatment set of CT images (CTmid). These CT images were taken 1 week before and 3.5 weeks after starting RT, respectively (Figure 1).

### 2.3. Radiomics Feature Extraction and Clinical Features

For radiomics analyses, regions of interest (ROIs) of the largest LN were drawn on CTpre and CTmid after matching the same lymph node. If the largest LN was conglomerated with circumferential LNs without a distinct border, the ROIs included all adjacent LNs. A radiation oncologist with over 20 years of experience performed 3D ROI segmentation using a semi-automated method (MRIcro).

A total of 70 radiomics features were extracted from each CT image. These features were divided into the following four categories: (1) histogram-based features (*N* = 19), which were computed using the voxel intensity of the tumor; (2) shape-based features (*N* = 11), which were calculated based on 2D and 3D ROIs; (3) texture-based features (*N* = 13), which were computed using GLCM (gray-level co-occurrence matrix) and GLSZM (gray-level size zone matrix); and (4) filtered-based features (*N* = 27), which were calculated using 3D Laplacian of Gaussian. A total of 70 radiomics features were extracted using a combination of PyRadiomics (ver. 3.0.)- and MATLAB (Math Works, Inc., Portola Valley, CA, USA)-based in-house code. Detailed descriptions of all features are given in Supplementary Table S1.

Clinical information on age, sex, primary site, smoking history, viral infection history, and disease stage according to the 7th AJCC stage were collected. In addition, specific information for the main LN such as size, degree of response during treatment, extracapsular extension (ECE), central necrosis (CN), multiplicity, bi-laterality, and level of lymph nodes was examined.

### 2.4. Radiomics Feature Selection

Radiomics analysis involves selecting features from the extracted feature set to effectively explain the intended clinical variables of interest. We applied the LASSO (Least Absolute Shrinkage and Selection Operator) method to select features associated with LN control. We applied cross-validation to the LASSO method for feature selection. After ten repetitions of the LASSO analysis, we selected features that were chosen five or more times as the final set (signature).

### 2.5. Statistical Test

**Figure 2:**
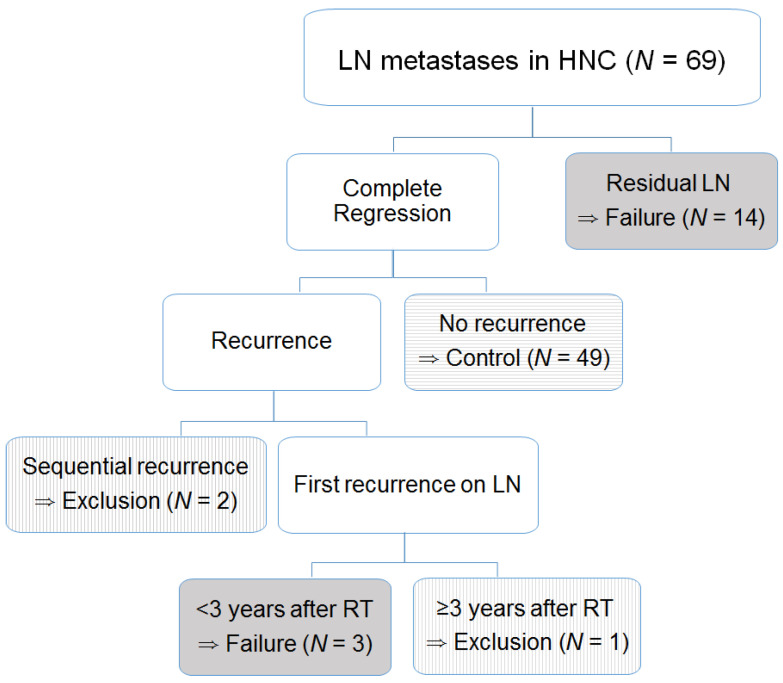
The flow of the selection process for the entire cohort. After excluding sequential recurrence following primary lesion and recurrence over 3 years (vertical line), a total of 17 LN control failures (gray color) were considered in 66 cases for radiomics analyses.

Our endpoint was the failure of LN control with residual or recurrent LN of an ROI after RT or CRT. Our study restricted the observation period to be 3 years considering median follow-up of disease-free patients. Residual disease was confirmed with pathologic findings of LNs or biopsy and clinical progression. If the residual LN was totally regressed in pathologic findings with a stable status continued during follow-up examination, it was defined as successful LN control. If the recurrence of ROIs sequentially progressed over a window period of 3 months after the primary recurrence or distant metastases, those cases were excluded from our radiomics analyses. If the ROI was aggravated over 3 years after the end of RT or CRT, it was excluded from our radiomics analyses (Figure 2).

Survival was evaluated on the last follow-up day or event occurrence from the start of RT. All survival rates were calculated with Kaplan–Meyer methods.

The selected final signature set was input into a Random Forest (RF) model to predict LN control. We used 200 decision trees, which were trained using the training set and evaluated using the test set. The training and test sets were split at a 7:3 ratio. Patients were randomly selected for each iteration, and we repeated the performance tests 20 times. We measured AUC (area under the curve), sensitivity, specificity, and accuracy for objective performance evaluation. All statistical analysis procedures were performed using MATLAB.

We compared predictive performances using the same method across a total of five categories: CTpre, CTmid, clinical parameters alone, CTpre plus clinical parameters, and CTmid plus clinical parameters.

## 3. Results

### 3.1. Survival and Failure of LN Control

**Figure 3:**
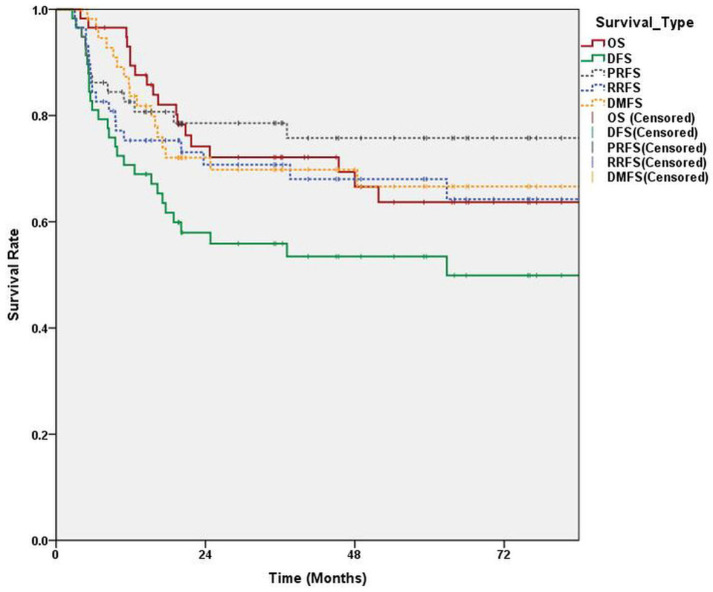
Survival curve of our cohort. OS: overall survival; DFS: disease-free survival; DMFS: distant metastases-free survival; RRFS: regional recurrence-free survival; PRFS: primary recurrence-free survival.

A total of 69 patients were analyzed. Table 1 provides detailed characteristics of the patients. Three-year disease-free survival and overall survival (standard error) were 53.3% (6.2%) and 73.2% (5.4%), respectively (Figure 3). Progression of primary site and distant metastases occurred in 15 and 20 patients, respectively. Among 21 patients with failure of LN control, two cases of LN recurrence were developed after 11 and 18 months of primary site recurrence and one case was developed 61 months after CRT. After excluding these three patients from the radiomics analyses, a total of 66 patients were enrolled with 17 LN control failures.

### 3.2. Feature Selection

In the CTpre alone model, out of a total of 75 radiomics features, two features were selected: size zone variance (GLSZM-based) and log minimum 2 (filtered-based). In the CTmid alone model, five features were selected: uniformity (histogram-based), volume (shape-based), energy (GLCM-based), homogeneity (GLCM-based), and log minimum 1 (filtered-based). In the clinical parameters alone model, age, virus status, LN size, ECE, and CN were important. In the CTpre plus clinical parameters model, out of 96 features including clinical parameters and radiomics features, 8 features were selected: spherical disproportion (shape-based), size zone variance (GLSZM-based), and log minimum 2 (filtered-based) of radiomics features and age, smoking, T-stage, ECE, and regression rate of LN. In the CTmid plus clinical parameters model, a total of eight features were selected: volume (shape-based), energy (GLCM-based), homogeneity (GLCM-based), log minimum 1 (filtered-based), smoking, T-stage, ECE, and regression rate of LN.

### 3.3. Performance Test

**Figure 4:**
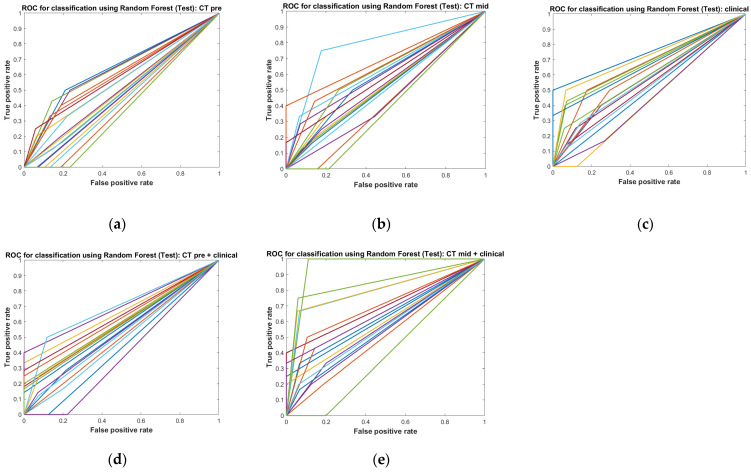
Visualization of receiver operating characteristic (ROC) curves. Each line represents the visualization of an ROC curve during the performance test, with a total of 20 repetitions. Each color line means the outcome of independent performance test. Each figure corresponds to (**a**) the CT_pre_ alone model, (**b**) the CT_mid_ alone model, (**c**) the clinical parameters alone model, (**d**) the CT_pre_ plus clinical parameters model, and (**e**) the CT_mid_ plus clinical parameters model.

The predictive model of CTpre and CTmid consisted of two and five different radiomics features, respectively. Clinical parameters including smoking, T-stage, ECE, and diameter regression rate during RT were commonly used for both models. Age was additionally used for the CTpre model. The mean accuracies (standard deviation) of the models with CTpre, CTmid, and clinical parameters were 0.681 (0.069), 0.698 (0.089), and 0.726 (0.089), respectively (Figure 4). These mean values of CTpre and CTmid plus clinical parameters became 0.731 (0.100) and 0.790 (0.095), respectively. Mean areas under the curve (AUC) of the models with CTpre, CTmid, and clinical parameters were 0.521 (0.080), 0.568 (0.093), and 0.593 (0.085), respectively. These mean values of CTpre and CTmid became 0.582 (0.088) and 0.662 (0.133) when the clinical parameters were included. These results are summarized in Table 2.

### 3.4. Feature Importance

**Figure 5:**
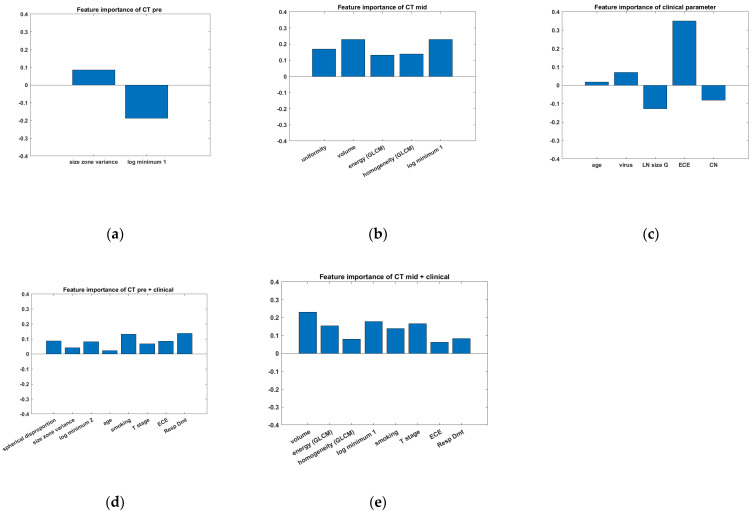
Visualization of the importance of each feature calculated using out-of-bag with a histogram. Each figure corresponds to (**a**) the CT_pre_ alone model, (**b**) the CT_mid_ alone model, (**c**) the clinical parameters alone model, (**d**) the CT_pre_ plus clinical parameters model, and (**e**) the CT_mid_ plus clinical parameters model that underwent performance testing using the out-of-bag method.

We evaluated the importance of features for each model that underwent performance testing using the out-of-bag method. The importance of each feature is visualized in Figure 5.

## 4. Discussion

This study aimed to suggest a model to predict LN control failure after definite RT in advanced HNC with regional LN metastases. It was designed to determine whether radiomics differentiate the intrinsic sensitivity of LN to radiation doses and whether clinical parameters have a synergic effect over radiomics alone. LNs were targeted as ROIs in our study. Since LNs have a round or oval shape different from a primary tumor, the physician could more readily conduct ROI delineation. Another benefit is the reproducibility of CTmid images since LNs maintain a consistent shape despite regression during treatment. Lastly, as shown in the recurrence pattern of our study, the LN was a common first recurrence site in locally advanced HNC and an important factor in determining the success of definite RT. The radiomics alone model for both CTpre and CTmid showed a moderate ACC to predict LN control failure. The predictability of radiomics was improved after adding clinical parameters.

A previous radiomics study of LN regression for 374 LNs from 113 patients showed an AUC of 0.71 in external validation [12]. Although our study differed from the above study in that only the largest lymph node was selected and analyzed in each patient, the outcomes of our study fell short with an ACC of 0.698 and an AUC of 0.568 in the CTmid set. In addition, a high ECE rate (58.0%) showed that our cohort consisted of more advanced LN metastases.

In our study, node regression rates during RT, ECE, T-stage, and heavy smoking were significant clinical parameters that could intensify the accuracy and AUC of radiomics. Previous studies have found that clinical–radiomics models show improved predictability, similar to our study. The radiomics features from MRI combined with clinical information improved the predictability of DFS and OS [13]. One report showed that combining genetic information on the hedgehog pathway and E2F transcriptional targets can potentially improve a radiomics model [14]. For lung cancer, dosimetric parameters of stereotactic body RT could improve the predictability of local control in addition to the clinical–radiomics model [15]. Thus, the radiomics model could be improved by adding other fields or omics information related to prognoses.

In the CTpre alone and the CTpre plus clinical parameters models, size zone variance and log minimum (σ = 2) were consistently chosen. Size zone variance measures how diverse the sizes of identical texture regions are within the ROI, reflecting texture heterogeneity. The log minimum is primarily observed at a tumor’s edge and is sensitive to subtle texture changes within the tumor. In the CTmid alone and the CTmid plus clinical parameters models, volume, energy (GLCM-based), homogeneity (GLCM-based), and log minimum were consistently selected. Volume, representing the size of the tumor projected in the image, was deemed crucial for predicting LN failure in this study. Energy (GLCM-based), signifying the degree of brightness variation within an ROI, indicates texture heterogeneity, expressing a tumor’s complexity and contributing as an important predictive factor of LN failure. Homogeneity, similar to energy, measures a tumor’s consistency and assesses the tumor’s unseen uniformity, thus playing a significant role in predicting LN control failure. The log minimum (σ = 1), a feature common with CTpre, was chosen again, reinforcing its role as a predictive feature of LN control failure in this study. Our research results encompass various aspects such as tumor texture, size, and brightness changes. By considering these factors collectively, we anticipate enhancing the accuracy of the LN control failure.

In terms of TME changes during RT, the radiomics at about 40 Gy was another key point of our study. For TME in HNC, there have been studies performed to distinguish subtypes of HNC using radiomics. One study showed that radiomics analysis could identify biologic features of tumors such as HPV status and T-cell infiltration [16]. Another study using 12 radiomics features more efficiently differentiated HPV-positive tumors in HNC [17]. Radiomics was also useful in distinguishing atypical, basal, classical, and mesenchymal subtypes of HNC [14]. These research studies suggest that intrinsic TME during RT could be detected with radiomics. In our study, the radiomics of mid-treatment at 40 Gy was better than the radiomics at pretreatment, suggesting that intrinsic sensitivity to radiation could be more efficiently presented during RT. Due to the limitations of a retrospective study, changes were observed once in the fourth week during RT. Therefore, the optimal timing to observe radiation effects should be examined in further studies. In one study using 18F-FDG-PET/CT, the cluster of metabolic radiomics at 20 Gy showed a significant relation with recurrence-free survival in oropharyngeal cancer [18]. In another study using 18F-FMISO-PET to assess hypoxia of intra-tumor, radiomics at 2 weeks and 5 weeks showed higher predictability of the treatment response with an AUC of approximately 0.8 in HNC [19]. It would be necessary to combine metabolic images and enhanced CT images to improve the predictive model of intrinsic sensitivity.

Delta radiomics can be used to analyze paired images and observe changes in TME. In the case of tumors, it was difficult to control the image itself through deformation because regression during treatment occurred and the ROI was deformed. It was also hard to judge the appropriateness of the values of delta features by subtracting or fractionating from one to another. Therefore, delta radiomics was not used in our study. Normal tissues such as salivary glands would be beneficial to examine the change with delta radiomics because the contour of the ROI is preserved [20]. In nasopharyngeal cancer, delta radiomics has been attempted using MRI. Three image sets at pretreatment and after induction chemotherapy and CRT were used, and the AUC to predict the efficacy of definite therapy was improved with delta radiomics [21]. Using cone-beam CT to originally check inter-fractional variation during RT is another method applied in delta radiomics. The model using radiomics features including coarseness and hemoglobin level moderately predicted tumor response in HNC in delta radiomics of cone-beam CT [22]. However, since the image quality is lower than helical CT images, its practical application still has limitations.

This study was based on retrospectively collected image data over about 10 years. Although the protocol for acquiring CT images for HNC has not changed, a few biases might have developed. Some artifacts from the immobilization device of aquaplast and head rest and prosthetics of teeth especially affected the LN in level IIa. This is an important problem that must be solved for radiomics studies using simulation CT images of RT. Efforts should be made to secure appropriate image quality in actual practice in future studies. Second, our study consisted of various kinds of primary sites originating from epithelial cells in HNC. Since it is important to analyze a certain number of patients as a preliminary radiomics study, examinations according to each sub-primary site were not performed. In addition, there were no data on HPV in some patients at the beginning of this study. Therefore, it was not sufficiently analyzed as an important clinical parameter. Based on this study, future research will be conducted targeting a more refined patient group by recruiting multiple institutions. Lastly, the RT technique was changed from 3DCRT to IMRT, although patients received sufficient radiation doses for ROIs regardless of the RT technique.

## 5. Conclusions

This preliminary study presented radiomics results of CT images for pretreatment and mid-treatment to predict LN control failure after definite RT in HNC with LN metastases. Both models were improved by adding clinical parameters. The model of CTmid plus clinical parameters was the best in our analyses. However, the results shown in our study still lacked predictive power to determine significant modification of treatment methods during definite RT. To activate radiomics research in HNC, efforts are needed to acquire high-quality images with minimum artifacts in actual clinical practice. In addition, future studies should combine various omics methods using other kinds of images, biomarkers, and genetic information.

## Figures and Tables

**Table 1:** Distribution of clinical parameters (*N* = 69).

Category	Sub-Category	
Sex	Male: female	58:11
Age (years)	Median (range)	55 (26–84)
Primary site	Nasopharynx:oropharynx:larynx:oral cavity: hypopharynx	25:31:3:1:9
Virus status	EBV:HPV:negative:unknown	21:17:11:20
Smoking	Never, ex-smoker, current smoker	24:17:28
	≤10:>10 (pack years)	30:39
Primary tumor		
T-stage	T1–2: T3–4	39:30
Size	≤2:2.1–4:>4 (cm)	15:35:19
Lymph node (LN)		
N-Stage	N1:N2–3	15: 54
Size	≤3:3.1–6:>6	27:38:4
Multiplicity	≤2:>2 (lymph node stations)	29:40
Laterality	Unilateral: bilateral	33:36
Extra capsular extension	No:yes	29:40
Central necrosis	No:yes	27:42
Total radiation dose	<70:≥70 (Gy)	9:60
Concurrent chemotherapy	No:yes	2:67
Regression of the largest LN size (long diameter CT_mid_/CT_pre_)	Median (range)	0.762 (0.436–1.250)

**Table 2:** Values (mean ± standard deviation) of ACC and AUC according to various radiomics models.

	CT_pre_	CT_mid_	Clinical Parameters	CT_pre_ Plus Clinical Parameters	CT_mid_ Plus Clinical Parameters
ACC	0.681 ± 0.069	0.698 ± 0.089	0.726 ± 0.089	0.731 ± 0.100	0.790 ± 0.095
AUC	0.521 ± 0.008	0.568 ± 0.093	0.593 ± 0.085	0.582 ± 0.088	0.662 ± 0.133

ACC: accuracy; AUC: area under the curve.

## Data Availability

The original contributions presented in this study are included in this article/Supplementary Material. Further inquiries can be directed to the corresponding authors.

## Supplementary Materials

The following supporting information can be downloaded at: https://www.mdpi.com/article/10.3390/medicina60010092/s1, Table S1: Detailed descriptions of all radiomics features.
