# Supplementary material for: A Potential Radiomics–Clinical Model for Predicting Failure of Lymph Node Control after Definite Radiotherapy in Locally Advanced Head and Neck Cancer
Source: Medicina (Kaunas). 2024 Jan 3;60(1):92. doi: 10.3390/medicina60010092 (PMC10821036; doi:10.3390/medicina60010092)
Supplement: Supplementary file 1 [file medicina-60-00092-s001.zip › medicina-2751471-supplementary.pdf]

Supplementary Table

Table S1. Definition of Radiomic Features

	**Parameter**	**Formula**	**Description**	**Image Biomarker Standardisation Initiative (IBSI) compliance**
**Histogram-based features**	Max, Min	$\text{Max} = Max(X(i))$ or $\text{Min} = Min(X(i))$ Where X denotes the 3d image matrix with N voxel.	Measures maximum or minimum intensity value of a histogram	O
	Median	$\text{Median} = \frac{\text{X(i)}}{2}$ Where X denote the 3d image matrix	Measures median intensity value of a histogram	O
	Mean	$\text{Mean} = \frac{1}{\text{N}}\sum_{\text{i}}^{\text{N}} \text{X(i)}$ Where X denote the 3d image matrix with N voxel.	Measures mean intensity value of a histogram	O
	Variance	$\text{Variance} = \frac{1}{\text{N}-1}\sum_{\text{i}=1}^{\text{N}} (\text{X(i)} - \bar{\text{x}})^2$	Measures squared distances of each value of a histogram from the mean	O
	Standard deviation	$\text{Std} = \left(\frac{1}{\text{N}-1}\sum_{\text{i}=1}^{\text{N}} (\text{X(i)} - \bar{\text{x}})^2\right)^{1/2}$ Where X denote the 3d image matrix with N voxel.	Measures amount of variation of a histogram.	O
	Energy	$\text{Energy} = \sum_{\text{i}}^{\text{N}} \text{X(i)}^2$ Where X denote the 3d image matrix with N voxel.	Measures squared magnitude value of a histogram	O
	Skewness	$\text{Skewness} = \frac{\text{E}(\text{x}-\mu)^3}{\sigma^3}$ Where μ is the mean of x, σ is the standard deviation of x, E is the expectation operator.	Measures asymmetry of a histogram.	O
	Kurtosis	$\text{Kurtosis} = \frac{\text{E}(\text{x}-\mu)^4}{\sigma^4}$ Where μ is the mean of x, σ is the standard deviation of x, E is	Measures "peakedeness" of a histogram (flatness of histogram)	O

		the expectation operator.		
	Root mean square	Root mean square $= \sqrt{\frac{1}{N}\sum_{n=1}^{N}\lvert X_n \rvert^2}$ Where X denote the 3d image matrix with N voxel.	Measures the square-root of the mean of the squares of the values of the histogram. This feature is another measure of the magnitude of a histogram	O
	Inter quartile range	$IQR = Q_3 - Q_1$ Where Q_3 denote the 3rd quartile of histogram, Q_1 denote the 1st quartile of histogram	Measures of variability, based on dividing a histogram into quartiles	O
	Range	Range = range(X(i))	Measures difference between the highest and lowest voxel values of a histogram	O
	Percentile	Percentile $= \left(\frac{n^{th}\ percentile}{100}\right)$ X(i)	Measures intensity value at the 2.5th, 25th ,50th ,75th, and 97.5th percentile on histogram	O
	Entropy	$\text{Entropy} = -\sum_{i=1}^{N_l} P(i)\log_2 P(i)$ Where P denotes the first-order histogram with N_l discrete intensity levels.	Measures irregularity of a histogram.	O
	Uniformity	$\text{Uniformity} = \sum_{i=1}^{N_l} P(i)^2$ Where P denotes the first-order histogram with N_l discrete intensity levels.	Measures uniformity of a histogram.	O
**Shape- based features (참고문헌 2개)**	Compactness	$\text{Compactness} = \frac{V}{\sqrt{\pi}A^{\frac{3}{2}}}$ Where V denotes the volume, and A denotes the surface area of the volume of interest (VOI)	Quantifies how close an object is to the smoothest shape, the circle	O
	Surface area	$SA = \sum_{i=1}^{N}\frac{1}{2}\lvert a_i b_i \times a_i c_i \rvert$ Where N is the total number of triangles (coved surface area), and a, b, c are edge vectors	The surface area of the ROI	O

	Convexity	$\text{Convexity} = \frac{V}{V'}$ Where V denotes tumor volume, and V' denotes convex hull volume	Measures ratio of the ROI volume contained within the tumor to the calculated convex hull volume	O
	Sphericity	$\text{Sphericity} = \frac{\pi^{\frac{1}{3}} \times (6V)^{\frac{2}{3}}}{A}$ Where A denotes area, and V denotes tumor volume	Measures of the roundness of the ROI	O
	Spherical disproportion	$\text{Spherical disproportion} = \frac{A}{4\pi R^2}$ Where R is the radius of a sphere with the same volume as the tumor	The ratio of the surface area of the ROI to the surface area of a sphere with the same volume as the ROI	O
	Maximum 3D diameter	See description in the next column	Measures of the maximum 3D ROI diameter. It is measured as the largest pairwise Euclidean distance, between surface voxels of the ROI	O
	Surface to volume ratio	$\text{Surface to volume ratio} = \frac{A}{V}$ Where A is area, and V is volume	Surface to volume ratio in ROI	O
	Volume	$\text{Volume} = \text{R} * \text{number of voxels}$ Where R denote the 3d image resolution	Volume of tumor (ROI)	O
	Roundness factor (2D)	$\text{Roundness factor} = \frac{4\pi \cdot Area}{Perimeter^2}$	Measure of circularity of a ROI	O
	Eccentricity (2D)	$\text{Eccentricity} = \text{c/a}$ Where c is the distance from the center to a focus and a is the distance from that focus to a vertex	Measure of how the tumor shape is close to the circle	O
	Solidity (2D)	$\text{Solidity} = \frac{Area}{Convex\ area}$	Measure of convexity of a ROI on the 2D image	O

**Texture - based features (GLCM)**	Autocorrelation	$\text{Autocorrelation} = \sum_{i=1}^{N_g}\sum_{j=1}^{N_g} ij\boldsymbol{P}(i,j)$	Measures of the magnitude of the fineness and coarseness of texture	O
	Cluster tendency	$\text{Cluster tendency} = \sum_{i=1}^{N_g}\sum_{j=1}^{N_g}[i + j - \mu_x - \mu_y]^2\mathbf{P}(i,j)$	Measures of the homogeneity of GLCM	O
	Maximum probability	$\text{Maximum probability} = \max\{P(i,j)\}$	Measures maximum value of GLCM matrix	O
	Contrast	$\text{Contrast} = \sum_{i=1}^{N_g}\sum_{j=1}^{N_g}\lvert i - j\rvert^2\mathbf{P}(i,j)$	Measures of the local intensity variation of GLCM	O
	Difference entropy	$\text{Difference entropy} = \sum_{i=0}^{N_g-1}\mathbf{P}_{x-y}(i)\log_2[P_{x-y}(i)]$	Measures entropy of processed GLCM matrix Px-y	O
	Dissimilarity	$\text{Dissimilarity} = \sum_{i=1}^{N_g}\sum_{j=1}^{N_g}\lvert i - j\rvert\mathbf{P}(i,j)$	Measures difference of each element of the gray level	O
	Energy	$\text{Energy} = \sum_{i=1}^{N_g}\sum_{j=1}^{N_g}[\mathbf{P}(i,j)]^2$	Measures of the homogeneity of GLCM	O
	Entropy	$\text{Entropy} = -\sum_{i=1}^{N_g}\sum_{j=1}^{N_g}\mathbf{P}(i,j)\log_2[\mathbf{P}(i,j)]$	Measures irregularity of gray level.	O
	Homogeneity	$\text{Homogeneity} = \sum_{i=1}^{N_g}\sum_{j=1}^{N_g}\frac{\mathbf{P}(i,j)}{1 + \lvert i - j\rvert}$	Measures closeness of gray-level.	O
	Informational measure of correlation	$\text{IMC} = \text{HXY} - \frac{\text{HXY1}}{\max\{HX, HY\}}$	Secondary measure of Homogeneity	O
	Variance	$\text{Variance} = \sum_{i=1}^{N_g}\sum_{j=1}^{N_g}(i - \mu_x)^2 P(i,j)$	Measures dispersion of the parameter values around the mean of the combinations of reference and neighborhood pixels	O

	Where $\mathbf{P}(i,j)$ is the gray level co-occurrence matrix for $(\delta = 1, \alpha = 0)$,<br>N_g is the number of discrete intensity value in the image,<br>N is the number of voxels in the ROI,<br>μ is the mean of $\mathbf{P}(i,j)$,<br>$p_x(i) = \sum_{j=1}^{N_g} \mathbf{P}(i,j)$ is the marginal row probabilities,<br>$p_y(i) = \sum_{i=1}^{N_g} \mathbf{P}(i,j)$ is the marginal column probabilities,<br>μ_x is the expected value of marginal row probability,<br>μ_y is the expected value of marginal column probability,<br>σ_x is the standard deviation of p_x,<br>σ_y is the standard deviation of p_y,<br>$p_{x+y}(k) = \sum_{i=1}^{N_g} \sum_{j=1}^{N_g} \mathbf{P}(i,j)$, $i + j = k, k = 2,3,\ldots,2N_g$,<br>$p_{x-y}(k) = \sum_{i=1}^{N_g} \sum_{j=1}^{N_g} \mathbf{P}(i,j)$, $\lvert i - j \rvert = k, k = 0,1,\ldots,N_g - 1$,<br>$HX = -\sum_{i=1}^{N_g} \mathbf{P}_x(i) \log_2[p_x(i)]$ is the entropy of $\mathbf{P}_x$,<br>$HY = -\sum_{i=1}^{N_g} \mathbf{P}_y(i) \log_2[p_y(i)]$ is the entropy of $\mathbf{P}_y$,<br>$HXY = -\sum_{i=1}^{N_g} \sum_{j=1}^{N_g} \mathbf{P}(i,j) \log_2[\mathbf{P}(i,j)]$ is the entropy of $\mathbf{P}(i,j)$<br>$HXY1 = -\sum_{i=1}^{N_g} \sum_{j=1}^{N_g} \mathbf{P}(i,j) \log(p_x(i)p_y(j))$			
**Texture - based features (GLSZM)**	Size-zone variability	Size zone variability $= \frac{1}{\Theta} \sum_{m=1}^{M} \left[\sum_{n=1}^{N} \boldsymbol{P}(m,n) \right]^2$	Variability in the size of ROI	O
	Intensity variability	Intensity variability $= \frac{1}{\Theta} \sum_{n=1}^{N} \left[\sum_{m=1}^{M} \boldsymbol{P}(m,n) \right]^2$	Variability in the intensity of ROI	O
	Where $\boldsymbol{P}(m,n)$ is the intensity size zone matrix<br>Θ represents the number of homogeneous areas in tumor,<br>M is the number of distinct intensity values,<br>N is the size of homogeneous area in the matrix $\boldsymbol{P}(m,n)$			
**Filter-based features (LoG) (참고문헌 1개 삽입)**	Mean	$\text{Mean} = \frac{1}{N} \sum_{i}^{N} G(i)$<br>Where G denote the filtered 3d image matrix with N voxel.	Measurement of mean of ROI image processed by LoG filter	O
	Max	$\text{Max} = Max(G(i))$<br>Where G denotes the filtered 3d image matrix with N voxel.	Measurement of max intensity value of ROI image processed by LoG filter	O

	Min	$\text{Min} = Min(G(i))$ Where G denotes the filtered 3d image matrix with N voxel.	Measurement of minimum intensity value of ROI image processed by LoG filter	O
	Median	$\text{Median} = \frac{G(i)}{2}$ Where G denote the filtered 3d image matrix	Measurement of median intensity value of ROI image processed by LoG filter	O
	Standard deviation	$\text{Standard deviation} = \left(\frac{1}{N-1}\sum_{i=1}^{N}(G(i)-\bar{G})^2\right)^{1/2}$ Where G denote the filtered 3d image matrix with N voxel.	Measurement of standard deviation of ROI image processed by LoG filter	O
	Skewness	$\text{Skewness} = \frac{E(G-\mu)^3}{\sigma^3}$ Where μ is the mean of G, σ is the standard deviation of G, E is the expectation operator.	Measurement of skewness of ROI image processed by LoG filter	O
	Kurtosis	$\text{Kurtosis} = \frac{E(G-\mu)^4}{\sigma^4}$ Where μ is the mean of G σ is the standard deviation of G, E is the expectation operator.	Measurement of kurtosis of ROI image processed by LoG filter	O
	Uniformity	$\text{Uniformity} = \sum_{i=1}^{N_l} P(i)^2$ Where P denotes the first-order histogram with N_l discrete intensity levels.	Measurement of uniformity of ROI image processed by LoG filter	O
	Entropy	$\text{Entropy} = -\sum_{i=1}^{N_l} P(i)\log_2 P(i)$ Where P denotes the first-order histogram with N_l discrete intensity levels.	Measurement of entropy of ROI image processed by LoG filter	O
	$G(x,y,z,\sigma) = I(x,y,z) * \frac{1}{\sigma(\sqrt{2\pi})^3} e^{-\frac{x^2+y^2+z^2}{2\sigma^2}}$ $\sigma = 0.5 - 3.5, 0.5$ increments, where I(x,y,z) is the image, and * denote convolution			

Note.—GLCM = gray-level co-occurrence matrix, GLSZM = gray-level size zone matrix, LoG = Laplacian of Gaussian.
